# Reply to X. Farré et al

**DOI:** 10.1200/GO.23.00015

**Published:** 2023-04-26

**Authors:** Andrew Vickers, Alejandro Berlin, Gladell Paner, Matthew Cooperberg, Howard Wolinsky, Scott Eggener

**Affiliations:** Andrew Vickers, PhD, Memorial Sloan Kettering Cancer Center, New York, NY; Alejandro Berlin, MD, Princess Margaret Cancer Centre, University Health Network, Toronto, Canada; Gladell Paner, MD, University of Chicago Medical Center, Chicago, IL; Matthew Cooperberg, MD, University of California, San Francisco, CA; Howard Wolinsky, MS, Editor, TheActiveSurveillor.com; and Scott Eggener, MD, Center for Data Intensive Science at the University of Chicago, Chicago, IL

We would like to thank Dr Farré et al for their interest in our work, specifically their timely reminder that academic discourse about prostate cancer is far too focused on what happens in high-income countries (HIC), thus missing the broader global perspective. However, we disagree with their conclusion that any moves toward redesignation of Gleason score 6 prostate disease (G6) to avoid the label of cancer should be shelved until further global data are available.

First, we are not aware of any data nor justification as to why clinical or biological characteristics of G6 disease might vary by income, ethnicity, nationality, or geography. We see no reason to perpetuate a harmful status quo^1^ on the basis of speculation that future data might subsequently provide a rationale for it, nor do we see how registry data might ultimately counter the foundation of our recommendation. Routine treatment of G6 in any setting should be seen as analogous to routine colectomy for colon polyps. To this point, we still await the first documented report of pure G6 (without higher grade elements), leading to metastases or death.

Second, the redesignation of G6 would likely serve as a lever for *increasing* screening and earlier detection programs in both HIC and low- to middle-income countries by lessening concerns for unnecessary overtreatment. Screening and early detection are sorely lacking in many regions of the world, where prostate cancer is a leading cause of death, manifesting at later stages when it is too late for curative-intent therapy. We favor advocating for increased investment, expanded access, and appropriate training as essential measures to address these gaps.^2^ None of these efforts seem mutually exclusive from potential G6 redesignation.

Third, HIC may have access to magnetic resonance imaging (MRI) and genomics, but it is a humbling reminder that surveillance for G6 has a 15-year risk of cancer-specific mortality of 0.1%^3^ from a cohort and era when these tools were not used routinely. We have concerns that MRI and genomics for men with G6 are more likely to lead to unnecessary treatment than save a life.

Fourth, avoidance of the term “cancer” does not appear to have any important implications for population registries and research. Registries could still record cases of erstwhile G6 (simply by its new name—perhaps a nonbenign noncancer term such as “acinar neoplasm,” as suggested by some pathologists [Paner et al, Adv Anat Pathol 2023, in press]), just as they record cases of breast ductal carcinoma in situ, which does not invade the basement membrane.

Fifth, Farré et al rehash several common but discredited arguments against redesignation. One is that because high-grade cancer can be missed when we sample the prostate, G6 should be called cancer. We find this line of thinking unusual: no other area in medicine do we so regularly justify calling something a disease on the basis of something that might be present but was not observed histologically, just in case the disease is there but we did not find it. Indeed, following this logic, we should start using the term “cancer” to describe high-grade prostatic intraepithelial neoplasia (HGPIN) or even benign prostate disease on the grounds that high-grade cancer could have been missed by prostate biopsies. The authors also point out G6 shares molecular features with more advanced cancers, but it also does with atypical intraductal proliferation, HGPIN, and normal prostate cells.

Finally, the concern of interobserver variability among pathologists is legitimate and will continue to be, regardless of a nomenclature change. We maintain our choices about language should be driven by the clinical reality of that entity and what is best for patients.

Farré et al claim that advocates for relabeling G6 recommend it “no longer be mandatorily diagnosed, treated, or actively monitored.” This is highly inaccurate and betrays a fundamental misunderstanding of our argument, which is that decisions about diagnosis, treatment, and monitoring of G6 should be driven by best evidence rather than unduly influenced by the emotional impact of the word “cancer.” All our authors, save one, have each spent their entire career screening, diagnosing, monitoring, treating, or researching prostate cancer. The remaining author has prostate cancer. Our plea is that public health would dramatically improve if a name change were ever to be implemented. We strongly agree research, advocacy, resources, and training should be a high priority anywhere in the world where access and quality are suboptimal. Nevertheless, we also stand by our conclusions that using a noncancer designation for G6 is justified progress and would do far more good than harm in every region of the world.

## References

[1] Farré X Mousavi SM Marcos-Gragera R et al A global perspective on the controversy of gleason score 6 prostate cancer reporting: the potential role of population-based cancer registries JCO Glob Oncol 10.1200/GO.22.00427 2023 10.1200/GO.22.00427 PMC10281400 37099738

[2] Vickers AJ Cooperberg MR Eggener SE Removing the designation of cancer from grade group 1 disease will do more good than harm Eur Urol83:304-306, 2023 10.1016/j.eururo.2022.10.001 36280498

[3] Atun R Jaffray DA Barton MB et al Expanding global access to radiotherapy Lancet Oncol 16 1153 1186 2015 26419354 10.1016/S1470-2045(15)00222-3

[4] Tosoian JJ Mamawala M Epstein JI et al Active surveillance of grade group 1 prostate cancer: Long-term outcomes from a large prospective cohort Eur Urol 77 675 682 2020 31918957 10.1016/j.eururo.2019.12.017

